# Complex molecular mechanisms cooperate to mediate histone deacetylase inhibitors anti-tumour activity in neuroblastoma cells

**DOI:** 10.1186/1476-4598-7-55

**Published:** 2008-06-12

**Authors:** Annick Mühlethaler-Mottet, Roland Meier, Marjorie Flahaut, Katia Balmas Bourloud, Katya Nardou, Jean-Marc Joseph, Nicole Gross

**Affiliations:** 1 Paediatric Oncology Research, Paediatric Department, University Hospital CHUV, CH-1011 Lausanne, Switzerland; 2 Life Sciences Division, Lawrence Berkeley National Laboratory, University of California, Berkeley, CA 94720, USA; 3 Paediatric Surgery, Paediatric Department, University Hospital CHUV, CH-1011 Lausanne, Switzerland

## Abstract

**Background:**

Histone deacetylase inhibitors (HDACi) are a new class of promising anti-tumour agent inhibiting cell proliferation and survival in tumour cells with very low toxicity toward normal cells. Neuroblastoma (NB) is the second most common solid tumour in children still associated with poor outcome in higher stages and, thus NB strongly requires novel treatment modalities.

**Results:**

We show here that the HDACi Sodium Butyrate (NaB), suberoylanilide hydroxamic acid (SAHA) and Trichostatin A (TSA) strongly reduce NB cells viability. The anti-tumour activity of these HDACi involved the induction of cell cycle arrest in the G2/M phase, followed by the activation of the intrinsic apoptotic pathway, via the activation of the caspases cascade. Moreover, HDACi mediated the activation of the pro-apoptotic proteins Bid and Bim_EL _and the inactivation of the anti-apoptotic proteins XIAP, Bcl-x_L_, RIP and survivin, that further enhanced the apoptotic signal. Interestingly, the activity of these apoptosis regulators was modulated by several different mechanisms, either by caspases dependent proteolytic cleavage or by degradation via the proteasome pathway. In addition, HDACi strongly impaired the hypoxia-induced secretion of VEGF by NB cells.

**Conclusion:**

HDACi are therefore interesting new anti-tumour agents for targeting highly malignant tumours such as NB, as these agents display a strong toxicity toward aggressive NB cells and they may possibly reduce angiogenesis by decreasing VEGF production by NB cells.

## Background

Histone deacetylase inhibitors (HDACi) are promising new anti-tumour agents due to their low toxicity toward normal cells and their ability to inhibit tumour growth in vivo. HDACi are currently under clinical trials and have activity in hematologic malignancies and solid tumours at doses that are well tolerated by patients [1-3].

HDACs regulate the expression and the activity of many proteins involved in cancer initiation and progression. HDACs affect gene expression by deacetylation of histones and transcription factors, and also deacetylate numerous other cellular proteins involved in cell growth, cell migration, apoptosis and differentiation [2,4,5]. Thus HDACi mediate their anti-tumour action by transcription-dependent and transcription-independent mechanisms. HDACi induce diverse responses in tumour cells, such as differentiation, cell cycle arrest, cell death by the activation of the intrinsic apoptotic pathway, the extrinsic apoptotic pathway, autophagic cell death, mitotic failure, senescence, and ROS facilitated cell death in tumour cells [2,5,6]. In addition, several HDACi affect tumour progression by their activities on angiogenesis and metastasis [4,5,7]. Indeed, some HDACi were shown to inhibit angiogenesis *in vitro *and *in vivo*, and to reduce the expression of pro-angiogenesis factors such as HIF-1α and VEGF [8,9]. Particular response to HDACi vary with tumour cell type, the HDACi used, and the treatment modalities [3]. For example, cell cycle arrest was described to occur in G1/S or in G2/M depending on the tumour cell types studied [5,10,11].

Neuroblastoma is the most common solid extracranial tumour in children and cause 15% of death from neoplasia in children [12,13]. Therefore, there is an urgent clinical need for new therapeutic strategies, with improved therapeutic potential. Several reports have described the ability of HDACi such as CBHA, PB or BL1521 to induce cell cycle arrest in G1/S phase and caspase-dependent apoptosis in some NB cell lines [14-18]. The release of Bax from Ku70 following acetylation of Ku70 was shown to mediate HDACi-induced apoptosis in NB cells [17]. Some studies have reported a reduction of NB tumour growth *in vivo *either on HDACi treatment alone or in combination with other treatment modalities [16,19-22]. We have previously reported that subtoxic doses of HDACi sensitised NB cells to TRAIL-induced cell death by a caspases-dependent increase in the pro- to anti-apoptotic proteins ratio [23].

The present study dissects the detailed mechanisms of HDACi anti-tumour activity in NB cells, that revealed to be similar in both the S-type and N-type NB cells, in contrast to the chemotherapeutic drug Doxorubicin [24]. The three HDACi, sodium butyrate (NaB), Trichostatin A (TSA) and suberoylanilide hydroxamic acid (SAHA) induced a cell cycle arrest in G2/M phase, followed by induction of the intrinsic apoptotic pathway, via the activation of the caspases cascade. Interestingly, HDACi increased the ratio between pro- to anti-apoptotic proteins by different mechanisms, either by caspases dependent cleavage or by degradation via the proteasome pathway. In addition, hypoxia-mediated VEGF secretion by NB cells was reduced by HDACi.

## Results

### NaB, SAHA, and TSA induce cell cycle arrest in G2/M phases in NB cells

**Figure 1:**
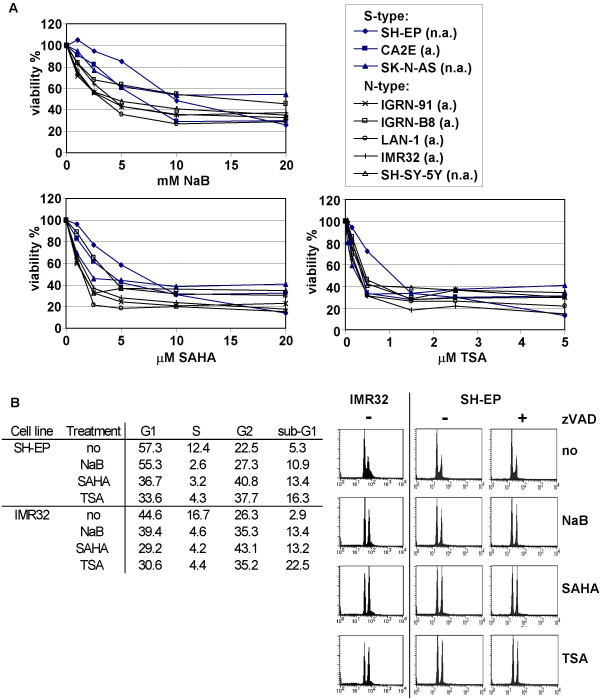
**NaB, SAHA, and TSA induce cell cycle arrest in G2/M phases in NB cells**. A. S-type and N-type NB cell lines were treated with increasing doses of NaB, SAHA or TSA as indicated for 48 h. Cell viability was measured by MTS/PMS cell proliferation assays. Mean values of three independent experiments are shown. MYCN non amplified (n.a.) and MYCN amplified (a.) cells are indicated. B. Cell cycle arrest was detected by the propidium staining method after stimulation for 16 h with 10 mM of NaB, 2.5 μM of SAHA or 1.5 μM of TSA for IMR32 cells, and with 20 mM of NaB, 20 μM of SAHA or 5 μM of TSA in the presence (+) or absence (-) of zVAD-fmk for SH-EP cells. Percentage of cells in G1, S, G2, and sub-G1 phases are indicated.

The anti-tumour activity of three HDACi, NaB, SAHA, and TSA was first analysed in various S-type and N-type NB cells. We observed that these HDACi reduced NB cell viability in a dose-dependent manner (Fig. 1A). While SH-EP cells were the less sensitive NB cells, no significant differences in the sensitivity to HDACi could be observed between S-type and N-type cells or between MYCN amplified compared to MYCN single-copy cells (Table 1).

**Table 1:** The sensitivity of NB cell lines to NaB, SAHA and TSA is independent of MYCN and p53 status.

cell line	IC_50 _NaB (mM)	IC_50 _SAHA (uM)	IC_50 _TSA (uM)	MYCN status	p53
SH-EP	11.1 ± 2.5	6.3 ± 3.0	1.3 ± 0.9	s.c.	wt
CA2E	5.9 ± 1.5	4.5 ± 3.0	0.4 ± 0.1	A	N.D.
SK-N-AS	13 ± 2.9	2.3 ± 0.3	0.3 ± 0.2	s.c.	mt^[45],[46]^
LAN-1	3.3 ± 1.1	1.4 ± 0.3	0.3 ± 0.2	A	mt^[47]^
IMR32	4.0 ± 1.4	2.5 ± 1.8	0.3 ± 0.2	A	wt^[47]^
SH-SY-5Y	5.0 ± 2.8	1.6 ± 0.6	0.4 ± 0.1	s.c.	wt
IGRN-91	3.8 ± 1.0	1.6 ± 0.4	0.7 ± 0.45	A	mt^[48]^
IGRN-B8	14.4 ± 2.2	3.9 ± 0.2	0.5 ± 0.1	A	mt^[46]^

s.c., single copy; A, amplified; wt, wild type; mt, mutated; N.D., not described

As HDACi are known to affect both cell survival and cell cycle progression, the effect of NaB, SAHA and TSA on cell cycle progression in NB cells was then measured in SH-EP, IMR32, LAN-1 and IGR-N91 cells. After 16 h of treatment, HDACi induced an accumulation of cells with 4n DNA content indicating a G2/M cell cycle arrest (Fig. 1B, and data not shown). In addition, the number of cells in S-phase was reduced following HDACi treatments (Fig. 1B). Cell cycle arrest was not inhibited by the addition of the pan-caspase inhibitor zVAD as illustrated with SH-EP cells (Fig. 1b). Thus, NaB, SAHA and TSA induce a caspases independent cell cycle arrest in G2/M phase in both S- and N-type NB cells.

### HDACi induce caspases-dependent cell death

**Figure 2:**
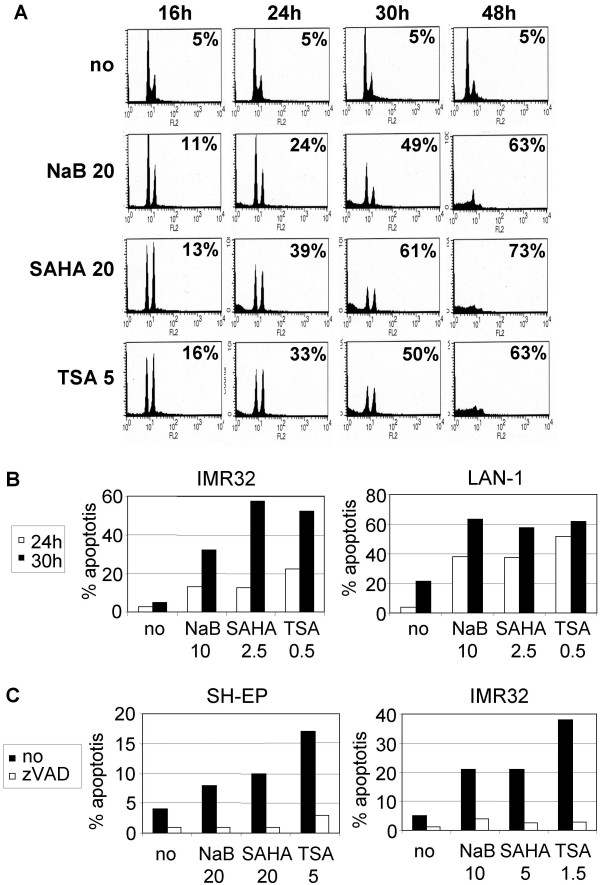
**Caspases-dependent apoptosis is mediated by HDACi**. Time-course analyses of HDACi-induced apoptosis in SH-EP cells (A), in IMR32 and LAN-1 cells (B). Cells were treated with indicated doses of NaB (mM), SAHA (μM) or TSA (μM). C. The caspases inhibitor zVAD completely prevents HDACi-induced apoptosis. SH-EP and IMR32 cells were treated for 24 h with NaB, SAHA, or TSA, in the presence (white) or in the absence (black) of zVAD. Percentage of sub-G1 apoptotic cells detected by the propidium staining method after stimulation with HDACi are indicated in the histograms (A), or as bare graph (B and C).

HDACi-mediated apoptosis induction was then investigated by the propidium iodide staining method in SH-EP, LAN-1 and IMR32 cells. The percentage of sub-G1 population of cells corresponding to apoptotic cells increased over time between 24 h to 48 h (Fig. 2A and 2B). Apoptotic cell death induced by HDACi was completely protected by the pan-caspases inhibitor zVAD (Fig. 2C). These data indicate that HDACi induce caspases-dependent cell death in both S- and N-type NB cells, and apoptosis occurs following cell cycle arrest in G2/M phase.

### HDACi activate the intrinsic apoptotic pathway

**Figure 3:**
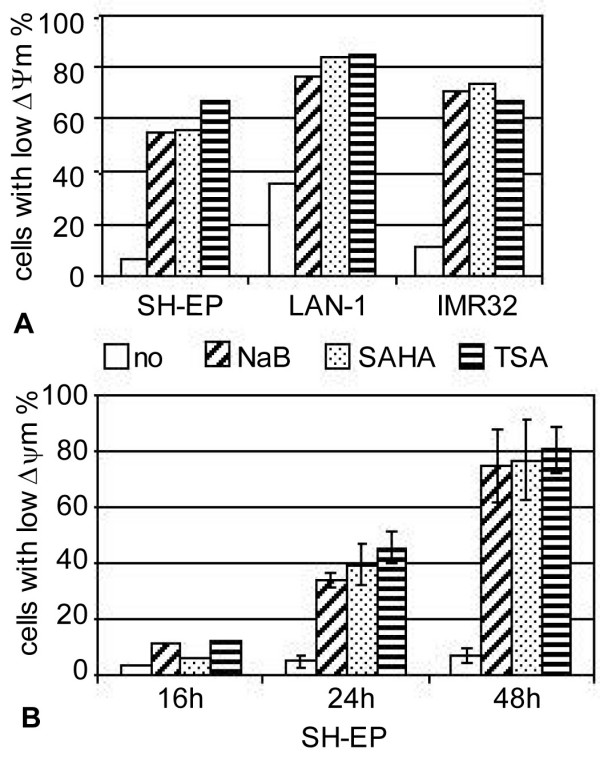
**Activation of the mitochondrial signalling pathway**. A. SH-EP, LAN-1, and IMR32 cells were untreated (no) or treated with NaB, SAHA or TSA for 48 h as indicated in Fig. 2A and 2B. B. SH-EP cells were treated with NaB, SAHA or TSA (as in Fig. 2A) for 16, 24, or 48 h. The loss of ΔΨm was measured by flow cytometry using the fluorescent dye JC-1. The percentage of cells with low ΔΨm is indicated. Mean of two independent experiments are indicated in B.

To analyse if HDACi induce apoptosis by activating the mitochondrial pathway, we measured the disruption of the transmembrane mitochondrial potential (ΔΨm) (Fig. 3A). Treatment of SH-EP, LAN-1 and IMR32 cells either with NaB, SAHA or TSA induced a strong reduction of the ΔΨm in 60% to 80% of these cells after 48 h of treatment. Time-course experiments showed that the disruption of the ΔΨm was enhanced between 24 h to 48 h (Fig. 3B), which correlates with the timing of apoptosis induction observed in figure 2A and 2B. The depolarisation of the membrane is partially caspases dependent, as zVAD moderately protects from reduction of the ΔΨm (data not shown). These results indicate that HDACi induce the activation of the intrinsic apoptotic pathway in NB cells.

### HDACi activate the caspases cascade

**Figure 4:**
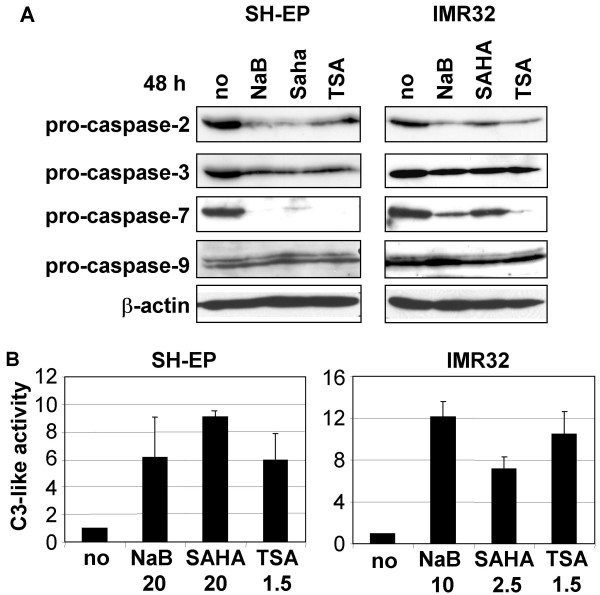
**HDACi activate the caspases cascade**. A. Cells were untreated (no) or treated with 20 mM NaB, 20 μM SAHA or 5 μM TSA for SH-EP, or with 10 mM of NaB, 2.5 μM of SAHA or 1.5 μM of TSA for IMR32 during 48 h. Whole cell extracts were analysed by immunoblotting for the cleavage of caspases-2, -3, -7 and -9. β-actin was used as loading control. B. Caspase-3-like activities are induced by HDACi. Hydrolysis of DEVD-pNA was measured in SH-EP or IMR32 cells unstimulated (no) or stimulated with NaB (mM), SAHA (μM), or TSA (μM) for 48 h as indicated. The caspase-3-like activities of stimulated cells, relative to unstimulated cells are indicated.

As apoptosis induced by HDACi was protected by zVAD, this suggests that HDACi activate the caspases cascade. Indeed, immunoblotting analyses revealed that NaB, SAHA and TSA induce the reduction of procaspases-2, -3 and -7 in SH-EP and IMR32 cells after 48 h of treatment, while procaspase-9 was unaffected (Fig. 4A). As apoptosis was shown to be dependent of caspases activity (Fig. 2C), the reduction of pro-caspases induced by HDACi is due to their activation by proteolytic cleavage rather than other down-regulation mechanisms. Time-course analysis of caspases cleavage indicated that it occurred between 16 h to 30 h (data not shown). To confirm the caspases activation mediated by HDACi, we measured caspase-3-like activity in SH-EP and IMR32 cells using the DEVD-pNA substrate. Caspase-3-like activity was induced in cells treated with NaB, SAHA, and TSA (Fig. 4B), indicating that these HDACi induce caspases activation in both S- and N-type NB cells.

### HDACi inactivate anti-apoptotic proteins by distinct mechanisms

**Figure 5:**
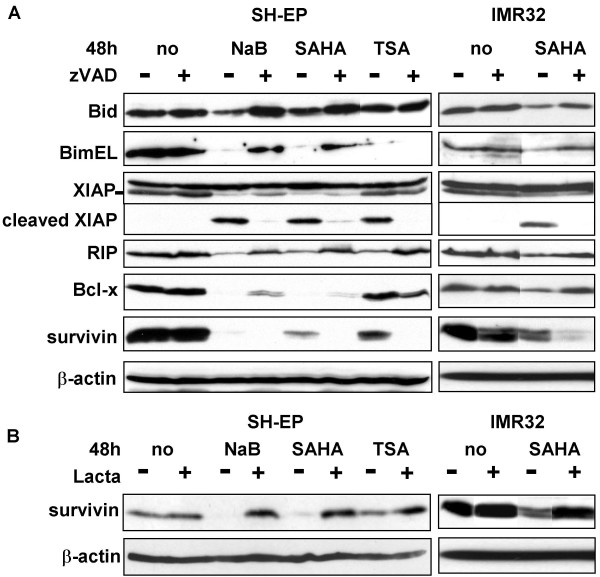
**HDACi increase the pro- to anti-apoptotic protein ratio by different mechanisms**. A. SH-EP cells were unstimulated (no), or stimulated with NaB (20 mM), SAHA (20 μM) or TSA (5 μM) in the absence (-) or in the presence (+) of zVAD-fmk for 48 h. IMR32 cells were unstimulated (no) or stimulated with 2.5 μM of SAHA in the absence (-) or in the presence (+) of zVAD-fmk for 48 h. Whole cell extracts were analysed by immunoblotting with anti-Bid, Bim, XIAP, RIP, Bcl-x_L _survivin antibodies. B. SH-EP cells were unstimulated (no), or stimulated with NaB, SAHA or TSA in the absence (-) or in the presence (+) of Lactacystin (10 μM) as in (A). IMR32 were unstimulated (no) or stimulated with SAHA (5 μM) in the absence (-) or in the presence (+) of Lactacystin (10 μM) for 48 h. Whole cell extracts were analysed for the presence of survivin. β-actin was used as loading control (A and B).

We have previously shown that subtoxic doses of HDACi in combination with TRAIL induced the inactivation of the anti-apoptotic proteins RIP, XIAP, Bcl-x_L_, and survivin, as well as the activation of Bid and Bim_EL _in a caspases dependent manner [23]. To analyse if toxic doses of HDACi induce similar effects, the expression level of these proteins were then investigated by immunoblotting. NaB and SAHA induced the reduction of the steady state level of the anti-apoptotic proteins XIAP, Bcl-x_L_, RIP, and survivin, and that of the pro-apoptotic proteins Bid and Bim_EL_, as observed in SH-EP cells (Fig. 5A) and in IMR32 cells (Fig. 5A and data not shown). These effects were observed between 16 h to 30 h of treatments (data not shown) and all except survivin were caspases-dependent as they were protected by the addition of zVAD (Fig. 5A). Indeed, zVAD did not protect the HDACi-mediated decrease in survivin expression, but on the opposite further enhanced survivin downregulation (Fig. 5A). TSA induced the same effect on XIAP, RIP, and survivin as NaB and SAHA. In contrast, Bid and Bcl-x_L _were not affected by TSA and the reduction of Bim_EL _was not protected by zVAD following TSA treatment.

We next investigated whether the reduction in survivin expression level mediated by HDACi results from its degradation by the proteasome using the proteasome inhibitor Lactacystin. Results shown in figure 5B indicate that Lactacystin strongly protected from a HDACi-mediated survivin down-regulation. This indicates that in NB cells, HDACi induce proteasome-mediated degradation of survivin, rather than caspases-dependent cleavage, as previously observed in co-treatment with subtoxic doses of HDACi and TRAIL [23].

### HDACi reduce hypoxia-induced secretion of VEGF

**Figure 6:**
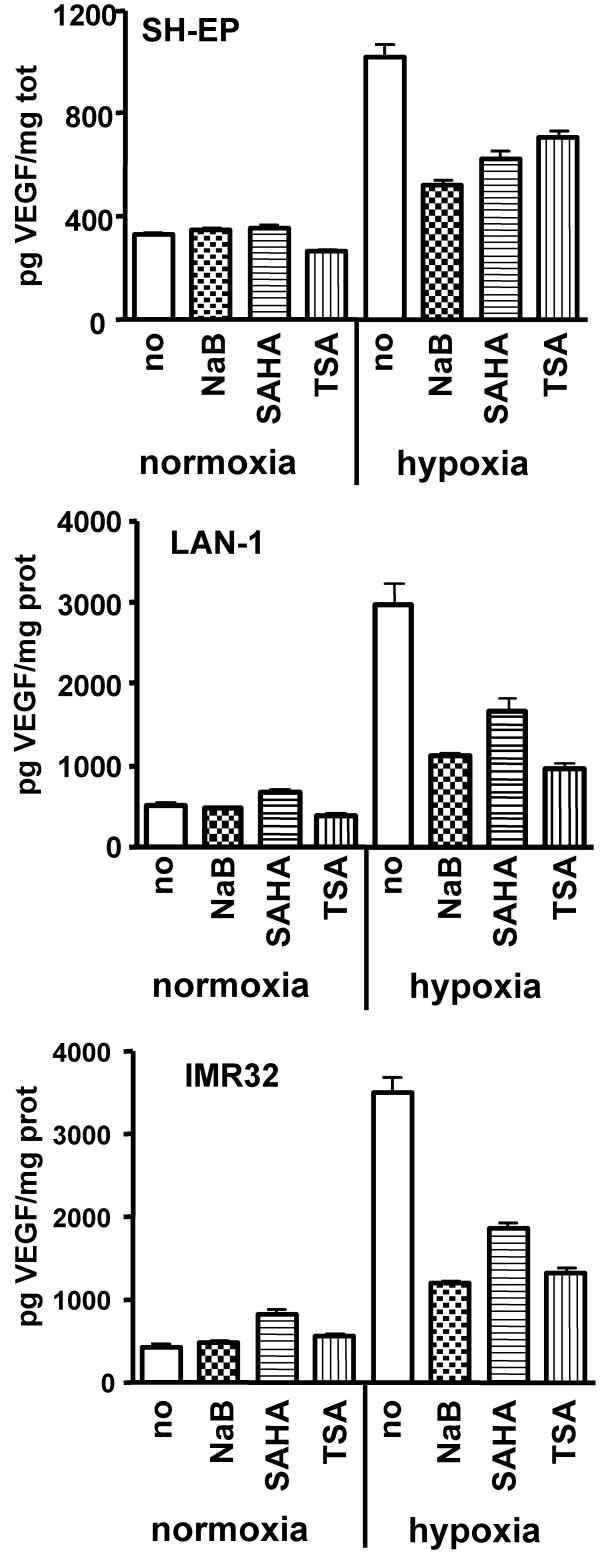
**HDACi repress hypoxia induced VEGF expression**. SH-EP cells were stimulated with NaB (20 mM), SAHA (20 μM) or TSA (5 μM), LAN-1 and IMR32 cells were stimulated with NaB (10 mM), SAHA (5 μM) or TSA (1.5 μM), or unstimulated (no) for 24 h under normoxia or hypoxia (1% O_2_). The bare graphs represent the amount of secreted VEGF in the supernatant normalized by the total amount of protein obtained in the corresponding cell lysate. Mean of duplicates of a representative experiment are indicated.

Certain HDACi mediate their anti-tumour activity by acting on cell cycle progression and survival, but also by affecting tumour angiogenesis via the reduction of HIF-1 and VEGF expression [4,5]. As NB are know to express high level of VEGF, we investigated whether NaB, SAHA and TSA had an effect on VEGF expression in NB cells. Results of ELISA assay show that the amount of secreted VEGF was strongly increased by hypoxia. Interestingly, NaB, SAHA and TSA significantly reduced hypoxia-mediated increase of VEGF secretion in both S-type (SH-EP) and N-type (LAN-1 and IMR32) NB cells (Fig. 6).

## Discussion

We have analysed in details the mechanisms of the antitumoral effect of the HDACi NaB, SAHA and TSA on human NB cells. All cell lines analysed were strongly sensitive to NaB (at mM concentration), or to SAHA and TSA (at microM concentration), as previously described for other tumour types [2]. With the exception of SH-EP cells, which were the less sensitive cells, all other cell lines displayed a similar sensitivity to the HDACi. Thus, no correlation could be found between the NMYC status, or the S-type versus N-type of the cells, and their sensitivity to HDACi. This is in agreement with a previous work reporting that the HDACi BL1521, TSA, and 4BP displayed a similar toxicity toward NMYC amplified and unamplified cells [25]. In contrast, other reports indicated that S-type cells were less sensitive to TSA than N-type cells [26], or that the NMYC amplified SMS-KCNR cells were more sensitive to MS-27-275 than the unamplified SK-N-AS cells [20]. In addition, in contrast to a very recent report showing that p53 is involved in the sensitivity of SH-SY5Y and SK-N-BE NB cell lines to VPA and Butyrate, we did not find any correlation between p53 status and NB cell lines sensitivity to NaB, SAHA and TSA (Table 1). These conflicting results may be explained by the fact that certain HDACi may exert their antitumoral activity by distinct mechanisms, as they selectively inhibit different HDACs [5,27]. Indeed, we have observed that TSA differently affected several pro- and anti-apoptotic proteins compared to NaB and SAHA.

Among the HDACi-activated antitumoral actions, cell cycle arrest has been described in many type of tumours [5,10,11], as well as in NB [15,16,18]. In contrast to a previous study indicating that the HDACI BL1521 induced cell cycle arrest in G1 phase in SK-N-AS and IMR32 cells [15], we have shown here that NaB, SAHA and TSA induce cell cycle arrest in the G2-M phase in both S-type and N-type NB cells. This is in accordance with a very recent report showing that Butyrate and VPA induce cell cycle arrest in the G2/M phase [28]. The cell cycle arrest induced by HDACi was observed around 16 h, preceding apoptosis induction, which occurs from 24 to 48 h. The cyclin-dependent kinase inhibitor p21 (WAF1/CIP1) is one of the most common target genes of different HDACi, and was reported to be largely associated with cell cycle arrest in G1 and G2 phases [5]. As previously described in NB cells [18,28], we also observed the overexpression of p21 (WAF1/CIP1) following treatment with NaB, SAHA and TSA (data not shown), thus p21 overexpression may play a role in the observed G2/M cell cycle arrest. In contrast, the cell cycle arrest was independent on p53, as several NB cells analysed express mutated p53 protein (Table 1).

We have shown here that NaB, SAHA and TSA induced caspases dependent apoptosis by the activation of the intrinsic apoptotic pathway in NB cells, as previously described in different tumours [10,11], and in NB cells for several HDACi [16,29]. In contrast, the involvement of TRAIL and the TRAIL-receptor pathway in HDACi-induced apoptosis was shown to vary depending on tumour cell types [30]. Our results demonstrated that in NB cell lines, apoptosis induced by NaB, SAHA and TSA did not required a functional death receptor pathway. Indeed, the N-type NB cell lines, that are deficient in the initiator caspases-8 and -10, displayed a sensitivity to HDACi similar to that of S-type NB cells expressing these caspases. In addition, the cell surface expression level of TRAIL and TRAIL-receptor was not increased by HDACi [23], and SH-EP cells overexpressing FLIP [31] displayed similar sensitivity to HDACi than control cells (data not shown). Altogether these findings indicate that the death receptor pathway is not involved in HDACi-induced apoptosis in NB cells, despite the fact that sub-toxic doses of HDACi can sensitise NB cells to TRAIL-mediated apoptosis [23]. Interestingly, no difference in the mechanisms of antitumoral response induced by HDACi could be observed between S-type and N-type NB cell lines. This is in contrast to the cytotoxic effect of Doxorubicin, which induced caspases-dependent cell death in S-type NB cells and caspases-independent cell death in N-type NB cells [24].

In this study we have further analysed in details the mechanisms of apoptosis induction by NaB, SAHA and TSA. The three HDACi increased the ratio between pro- and anti-apoptotic proteins. While NaB and SAHA mediated the downregulation of RIP, XIAP, Bcl-x_L _and survivin, and the activation of Bid and Bim_EL_, TSA only induced the downregulation of XIAP, RIP and survivin. Thus, different HDACi may act through distinct mechanisms to induce their antitumoral response in the same tumour cell type.

NaB and SAHA induced the caspases-dependent activation of Bid, as Bid reduction was protected by the pan-caspase inhibitor zVAD, which also occurs in N-type NB cells in absence of caspases-8 and -10 expression. This suggests that the activation of the intrinsic pathway proceeds via an amplification loop that is independent of caspases-8 and -10, but dependent of other caspases as disruption of the ΔΨm was partially protected by zVAD (data not shown). Bid was indeed reported to be the substrate of other caspases, such as caspase-2 or caspase-3 [32].

The activation of Bim_EL _by caspase-3 was previously shown to induce a positive amplification loop by increasing the affinity of Bim_EL _to Bcl-2 [33]. Thus, Bim_EL _may be activated by caspases-dependent cleavage by NaB and SAHA in NB cells, as previously observed by co-treatment with subtoxic doses of HDACi and TRAIL [23].

Interestingly, we have observed that the down-regulation of the anti-apoptotic proteins RIP, XIAP, Bcl-x_L _and survivin occurred by different mechanisms, either by caspases dependent cleavage or by degradation via the proteasome pathway. Indeed, in contrast to RIP, XIAP, and Bclx, the downregulation of survivin was not protected by the caspases inhibitor zVAD, which strikingly further increased survivin downregulation. In normal cells, survivin is expressed in a cell cycle-dependent manner, with an increased expression during the G2/M phase in normal cells [34] and survivin degradation is tightly regulated via the ubiquitin-proteasome pathway in a cell cycle dependent manner [35]. In tumour cells in contrast, survivin expression is globally deregulated and is overexpressed in all cell-cycle phases [36]. Thus, our observation that HDACi-mediated downregulation of survivin in NB cells despite G2/M phase cell cycle arrest was not surprising. Survivin downregulation was prevented by the proteasome inhibitor Lactacystin, indicating that NaB, SAHA and TSA mediated survivin degradation via the ubiquitin-proteasome pathway. This is in accordance with previous reports describing that the downregulation of survivin occurred via the proteasome pathway after induction with the HDACi chlamydocin in ovarian carcicoma cells [37] and with valproic acid in colon cancer [38]. Interestingly, the downregulation of survivin induced by subtoxic doses of HDACi in combination with TRAIL occurred by caspases-dependent cleavages [23]. Thus, the mechanisms of HDACi-mediated apoptosis may vary in the same cell lines depending on the doses or the addition of combined treatment. We have shown previously that the downregulation of survivin expression by siRNA sensitised NB cells to HDACi [23]. This suggests that part of the antitumoral effect of HDACi in NB cells may be due to the downregulation of survivin via the proteasome pathway.

In addition to the role of HDACi in reducing tumour initiation by their effects on cell proliferation and survival, HDACi were found to repress angiogenesis *in vitro *and *in vivo*, and to reduce the expression of pro-angiogenic factors, such as HIF and VEGF [5,8,9]. VEGF is a key regulator of angiogenesis, by inducing endothelial cell proliferation and migration [39]. Most NB cell lines and tumours express VEGF, and VEGF expression level correlates with tumour progression and poor prognostic [40,41]. Moreover, blockade of VEGF function was shown to be associated with suppression of NB growth [42,43]. We have shown here that NaB, SAHA and TSA strongly decrease the hypoxia-mediated induction of VEGF secretion in both S-type and N-type NB cells. This suggests that NaB, SAHA and TSA may be efficient to reduce NB growth *in vivo *by decreasing tumour angiogenesis in addition to tumour cell growth and survival. Indeed, HDACi-mediated reduction of VEGF expression by 4-phenylbutyrate, or decrease of tumour vasculature by MS-27-275 have been previously described in NB tumour xenograft model [16,20].

## Conclusion

By dissecting the molecular pathways involved in the HDACi-mediated toxicity in NB cells, we have identified the participation of a complex array of distinct mechanisms, that outline the complexity of the HDACi-mediated effects. In addition to the observation that NaB, SAHA and TSA are potent therapeutic agents against highly malignant NB cells, by affecting both cell cycle progression and survival, our study also underlines a possible antiangiogenic action of HDACi in NB. The effect of HDACi on VEGF production thus supports HDACi anti-tumour activity reported in human NB xenograft models [19,20] and suggests a role for the microenvironment, that should be rapidly addressed *in vivo*. Altogether, our and others observations confirm the interest in HDACi as promising new treatment modalities for patients with recurrent high-risk neuroblastoma.

## Methods

### Cell culture and reagents

The S-type NB cells SH-EP, CA2E, and SK-N-AS and the N-type NB cells LAN-1, IMR32, SH-SY-5Y, IGRN-91, IGRN-B8 were grown in DMEM medium supplemented with 2 mM L-glutamine, 100 U/ml Penicillin, 0.1 mg/ml streptomycin, and 10% of FCS. Sodium butyrate (Fluka) was dissolved in H_2_O and stored at -20°C. SAHA (Biovision) and TSA (Sigma) were dissolved in DMSO and store at -20°C. Cells were treated with the caspases inhibitor zVAD-fmk (100 μM, Bachem) or with Lactacystin (10 μM, Alexis Corporation) when indicated.

### Cell viability assays

Cells (1–2.5*10^4^/well in 96-well-plates; 100 μl) were plated 24 h before treatment and incubated with HDACi for 48 h. Assays were performed in quadruplicates. Viability was measured using the MTS/PMS cell proliferation kit from Promega according to manufacturer's instructions. Percentage of cell viability as compared to untreated controls was calculated.

### Apoptosis and cell cycle analyses by the propidium iodide staining method

Cells were washed twice with ice-cold PBS, resuspended in 1 ml of ice-cold PBS, and fixed with 3 ml of 100% ice-cold ethanol for 1 h at 4°C. For staining with propidium iodide (PI), cells were washed twice in ice-cold PBS, resuspended in 0.2 ml of PBS containing 200 μg/ml RNaseA and 10 μg/ml propidium iodide and incubated for 30 min at room temperature. The stained cells were analyzed using a FACScan flow cytometer (Becton Dickinson).

### Immunoblotting

Whole cell extracts were prepared as already described [44]. Protein extracts (50 μg) were loaded on 12% SDS-PAGE and transferred on nitrocellulose membranes. Blots were saturated with 5% skim milk, 0.1% Tween 20 in TBS and revealed using mouse monoclonal antibodies to detect caspase-2 (Apotech Corporation), caspase-3, caspase-7, RIP, XIAP (BD Transduction Laboratories), and β-actin (Sigma). Polyclonal rabbit antibodies were used to detect caspase-9 (Cell Signaling), Bid, Bcl-x_L _(BD Transduction Laboratories), Bim (Imgenex), and survivin (R&D systems). Binding of the first antibody was revealed by incubation with either goat anti-mouse IgG-HRP (Jackson ImmunoResearch) or goat anti-rabbit IgG-HRP (Nordic Immunological Laboratories). Bound antibodies were detected using the Lumi-light western blotting substrate (Roche) according manufacturer's instructions.

### Caspases activities

Caspase-3 like activities were measured using the caspases-3 colorimetric protease assay kit from MBL. Cytosolic lysates were prepared after HDACi treatments according to manufacturer instructions. Two hundred μg of protein extracts were incubated with 200 μM of DEVD-pNA colorimetric substrate for 3 h at 37°C. Cell lysates were incubated with 10 μM of caspase inhibitor (zDEVD-fmk) for 30 min before addition of caspase substrate to control the specificity of caspase-3 like activation. Hydrolysed pNA was detected using a microtiter plate reader at 405 nm. Background absorbance from cell lysates and buffers were subtracted from the absorbance of stimulated and unstimulated samples before calculation of relative caspases activities.

### Analysis of mitochondrial transmembrane potential

The disruption of mitochondrial membrane potential ΔΨm was analysed by staining the cells with the fluorescent dye JC-1 (Calbiochem) according to manufacturer's protocol. Loss of ΔΨm resulting in reduction of red aggregates was measured by flow cytometry using the FL2 channel (550–650 nm) (FACScan, Becton Dickinson). Results are given in percentage of cells with low ΔΨm compared to untreated controls.

### VEGF measurement

Cells (2.5*10^6 ^SH-EP. 3.5*10^6 ^LAN-1 and IMR32) were platted in 75 cm2 dishes the day before induction. Cells were treated with HDACi under normoxic condition or under hypoxia (1% O_2_) for 24 h. Cell free supernatant were collected and whole cell extracts were prepared by incubating cell pellets in lysis buffer (50 mM Tris-HCl pH 7.4, 150 mM NaCl, 10 mM EDTA, 0.25% Triton X-100, 0.1% NP-40) supplemented protease inhibitor cocktail tablets (*complete mini*, Roche) for 10 min in ice. Homogenates were centrifuged 20 min. at 16'000 g and supernatant were collected. Protein concentration was measured using the micro BCA protein assay kit (Pierce). Soluble VEGF was measured in 50 μl of supernatants in duplicates using the VEGF DuoSet ELISA kit (R&D system) according to manufacturer's instructions.

## Abbreviations

NB: Neuroblastoma; HDACi: histone deacetylase inhibitors; NaB: sodium butyrate; SAHA: suberoylanilide hydroxamic acid; TSA: Trichostatin A; TRAIL: Tumour Necrosis Factor-related apoptosis-inducing ligand; c-FLIP: cellular Flice inhibitory protein.

## Competing interests

The authors declare that they have no competing interests.

## Authors' contributions

AMM performed all major experimental work, participated in the design, the coordination of the study and drafted the manuscript, KBB participated in all cell culture experiments and performed the immunoblots, caspases activity assays, KA participated in VEGF measurement and in cells stimulation with drugs, MF developed the SH-EP FLIP cells, RM participated in cell treatments with HDACIs, JMJ and NG were involved in the overall design of the study and helped to draft the manuscript.
